# EEG individual power profiles correlate with tension along spine in horses

**DOI:** 10.1371/journal.pone.0243970

**Published:** 2020-12-14

**Authors:** Mathilde Stomp, Serenella d’Ingeo, Séverine Henry, Clémence Lesimple, Hugo Cousillas, Martine Hausberger

**Affiliations:** 1 Univ Rennes, Normandie Univ, CNRS, EthoS (Éthologie animale et humaine)—UMR 6552, Paimpont, France; 2 Department of Veterinary Medicine, Section of Animal Physiology and Behaviour, University of Bari “Aldo Moro”, Bari, Italy; La Sapienza University of Rome, ITALY

## Abstract

Assessing chronic pain is a challenge given its subjective dimension. In humans, resting state electroencephalography (EEG) is a promising tool although the results of various studies are contradictory. Spontaneous chronic pain is understudied in animals but could be of the highest interest for a comparative study. Riding horses show a very high prevalence of back disorders thought to be associated with chronic pain. Moreover, horses with known back problems show cognitive alterations, such as a lower attentional engagement. Therefore, we hypothesized that the individual EEG power profiles resting state (*i*.*e*. quiet standing) of different horses could reflect the state of their back, that we measured using static sEMG, a tool first promoted to assess lower back pain in human patients. Results show that 1) EEG profiles are highly stable at the intra-individual level, 2) horses with elevated back tension showed resting state EEG profiles characterized by more fast (beta and gamma) and less slow (theta and alpha) waves. The proportion of theta waves was particularly negatively correlated with muscular tension along the spine. Moreover, elevated back tension was positively correlated with the frequency of stereotypic behaviours (an “addictive- like” repetitive behavior) performed by the horses in their stall. Resting state quantitative EEG appears therefore as a very promising tool that may allow to assess individual subjective chronic pain experience, beyond more objective measures of tension. These results open new lines of research for a multi-species comparative approach and might reveal very important in the context of animal welfare.

## Introduction

The assessment of pain, and in particular chronic pain, is a major challenge that remains difficult to solve, especially since in addition to the nociceptive stimulation, there is a subjective emotional and cognitive dimension to it [1]. All authors agree that there is a need for a reliable readout for spontaneous pain, based on a numerical measure, and that would apply on awake unrestrained subjects of different species [2]. However most behavioural or even imagery paradigms (*i*.*e*. grimace scales, conditioned place preference, burrowing, brain imaging) proposed lately may be too subjective or lack the necessary temporal resolution to respond to these criteria [3].

Since pain reflects neural activity in the brain [2], there has been a growing interest in exploring resting state EEG oscillations as potential biomarkers in humans [3, 4]. Spectrum analysis can be used to assess resting state EEG signals in different frequency bands [5] and neuronal oscillations at different frequencies represent fundamental features of neuronal signalling [4]. Most studies performed with quantitative EEG, *i*.*e*. cortical wave power profiles [3], defined as an objective tool with high reliability and high clinical sensitivity that involves the use of computers and power spectral analyses [6], show a clear relation between frequency power (slow: delta, theta, alpha, or fast waves: beta, gamma) and the intensity of pain in humans [7, 8]. Differences in the F50, F95 and total power of the EEG were found in animals experiencing acute pain (sheep: [9]; lambs: [10]; calves: [11]; horses and ponies: [12]). However, results are somewhat contradictory (increase in theta versus gamma waves according to studies), which may be due to differences in procedures (spontaneous versus induced, acute versus chronic, objective/noxious versus subjective/emotional pain) [3, 4]. While quantitative EEG appears as a very promising tool, more studies on different species are needed in order to clarify its meaning.

In the present study, we used a telemetric EEG device to examine the possible relation between the relative distribution of the different EEG frequency bands and measures of back disorders (morphometry, muscular tension along the spine) in horses. Horses show a very high prevalence of back disorders and hence potential chronic back pain (35 to 100% of the ridden horses as shown through post-mortem anatomical data [13, 14], imagery [15], or palpation [16, 17]). The prevalence of back disorders in horses is not related to age [13, 18], but is associated with the type of work [15, 16]. Morphometric changes in back and neck shape (flattening of the spine, flat/hollow neck) [14, 19, 20] are associated with these differences [21–23]. Moreover, neck shape has been shown to differ between populations independently of sex, age or breed, reflecting working conditions and spine state [18, 22]. Back disorders in horses can result from various anatomical (impingement or overlap of dorsal spinuous processes, left/right spinal asymmetry) or muscular problems. Thus, the presence and degree of muscular asymmetry at the horse’s back level was significantly associated with osseous pathological changes, and potentially severe lesions showing active bone remodelling, *e*.*g*. [20]. Horses with observed back disorders are thought to experience chronic pain as their postural and behavioural (increased aggressiveness, lameness, avoidance of riding tack) characteristics strongly suggest [17, 19, 24, 25]. Static surface electromyography is a common measure of low back pain in humans with low back pain patients presenting higher sEMG levels than healthy controls [26, 27] and correlates with the evaluations of back disorders in horses by practitioners [22]. Excessive muscular activity is aggravated by nociception [28] and practitioners consider that elevated tenseness at vertebral sites reflects chronic pain [29, 30]. Horses with identified back disorders also show cognitive changes, in particular lowered attentional engagement [23]. Therefore, we hypothesized that back disorders would reflect in EEG profiles. An earlier study has shown that individual differences do exist in EEG profiles of horses recorded in a quiet standing state and that these profiles are stable over two separate sessions at least [31]. Therefore, we tested here whether there could be correlations between static sEMG (as an indicator of back state) and EEG (in terms of frequency band distribution) measures recorded independently on the same horses.

Finally, since aberrant addiction-like behaviours develop in almost a fourth of human patients with chronic back pain, often with opioid abuse [1], we also tested whether there was a relation between our measures of muscular activity and the presence and frequency of stereotypic behaviours, abnormal repetitive behaviours associated with endorphin releases. Horses’ stereotypic behaviour (defined as ‘repetitive behaviour induced by frustration, repeated attempts to cope and/or brain dysfunction’; *e*.*g*. [32]) and other abnormal repetitive behaviours are known to increase with chronic stress, and to constitute a reliable indicator of unfavourable conditions [33–36]. Stereotypic horses also show cognitive and reproductive impairments [37–39].

In order to test our hypothesis, 18 adult riding horses were submitted to EEG recordings using an ambulatory headset and a telemetric EEG recorder while being held standing quiet in a familiar covered arena. The relative EEG power values of delta (δ: 0–4 Hz), theta (θ: 4–8 Hz), alpha (α: 8–12 Hz), beta (β: 12–30 Hz) and gamma (γ: >30 Hz) bands were calculated for each horse. Such relative proportions have been shown to reliably reflect the attentional state of horses [40]. In parallel, their neck shape (as indicator of possible dorsal problems) was classified into flat/hollow versus round in accordance with Lesimple et al [22]’s morphometry measurements, which showed that horses with flat and hollow necks shared a tendency to have back disorders. Furthermore, thirteen of these horses could be submitted to sEMG recordings along the spine. We calculated the muscular activity’s median value of all vertebral sites along the spine per horse. In order to assess the degree of asymmetry we calculated the difference between the left and the right sides and obtained a median value of the global asymmetry along the spine per individual. We hypothesized that the spine state, which influences the affective state of the animal, would influence cognitive processing and thus brain waves production. In accordance with human studies, we predicted that: 1) since elevated/asymmetrical muscular activity along spine and a hollow/flat neck may reflect chronic back pain, these measures would be reflected in an overrepresentation of fast waves in the horses’ EEG profiles; 2) that, since stereotypic behaviours may be considered as addictive, they may be more frequent in horses with elevated muscular tension along the spine, suggestive of potential chronic pain, hence again with more fast waves.

All 18 horses were therefore also observed in their usual environment to assess the presence and frequency of stereotypic behaviours. This study is part of a larger project where relationships between life conditions, welfare state and cognitive processing are investigated [41]. Only the relationship between EEG profiles and back problems are described here on a subset of horses made available for this precise purpose. Comparisons of populations at a larger level are described elsewhere (Stomp et al. subm.).

## Materials and methods

### Ethical note

The experiments were carried out in 2017 in accordance with the European Parliament and the European Union Council relative to the animals’ protection used for scientific purposes directive 2010/63/UE and complied with the current French laws related to animal experimentation (decree n°2013–118 of 1 February 2013 and its five implementation orders (JO of 7 February 2013), integrated in the Code rural and the Code of the maritime fishing under n° R. 214–87 à R.214-137). According to these European and French laws, our experiment did not require an authorization request. Indeed, our manipulations did not cause any physical or mental pain, they consisted only of a common practice, which is the positioning of a helmet on a horse head that was held with a loose lunge in its home environment. Animal husbandry and care were under the management of the recreational horses’ owners.

### Subjects

The present study was conducted in July 2017 in Brittany (France) on a total of 18 individuals, 7 females, 7 geldings and 4 stallions, from various breeds (7 breeds represented, as well as unregistered or mixed-breed horses; 16.6% of French Saddlebreds) and ages (4 to 21 years old) (see Table 1). Horses lived on 2 sites where they had been living in the same conditions for at least one year.

**Table 1:** Characteristics of the individuals involved in the study.

Individuals Pop.1	Sex	Age	Individuals Pop. 2	Sex	Age
S1	Stallion	4	G5	Gelding	9
S2	Stallion	3	G6	Gelding	14
G1	Gelding	5	G7	Gelding	11
G2	Gelding	16	G8	Gelding	11
G3	Gelding	12	G9	Gelding	9
G4	Gelding	21	M4	Mare	14
M1	Mare	19	M5	Mare	12
M2	Mare	12	M6	Mare	10
M3	Mare	15	M7	Mare	12

The first site included nine recreational horses (N_mares_ = 3; N_stallions_ = 2; N_geldings_ = 4) from various ages (4 to 21 years old, X±SE = 13±6) and breeds. The animals lived under naturalistic conditions: all year long in 1–2 ha pasture with conspecifics, fed grass and hay *ad libitum* during winter (no industrial pellets) and were used for occasional outdoor relaxed leisure riding. In the second site, nine horses (N_mares_ = 4, N_geldings_ = 5, 9 to 14 years old, X +SE = 11.2+1.9, various breeds) were living in a riding school, and were kept in 3x3 m individual straw bedded stalls in a barn, were fed industrial pellets twice a day and hay (6-7kg) once a day, and were working in riding lessons for 4–12 hours per week under the supervision of a riding teacher. The horses were ridden with typical English riding style [18]. Horses went out to paddocks (with grass) every day from one to four hours per day.

### Electroencephalography (EEG) recordings & data analysis

**Fig 1:**
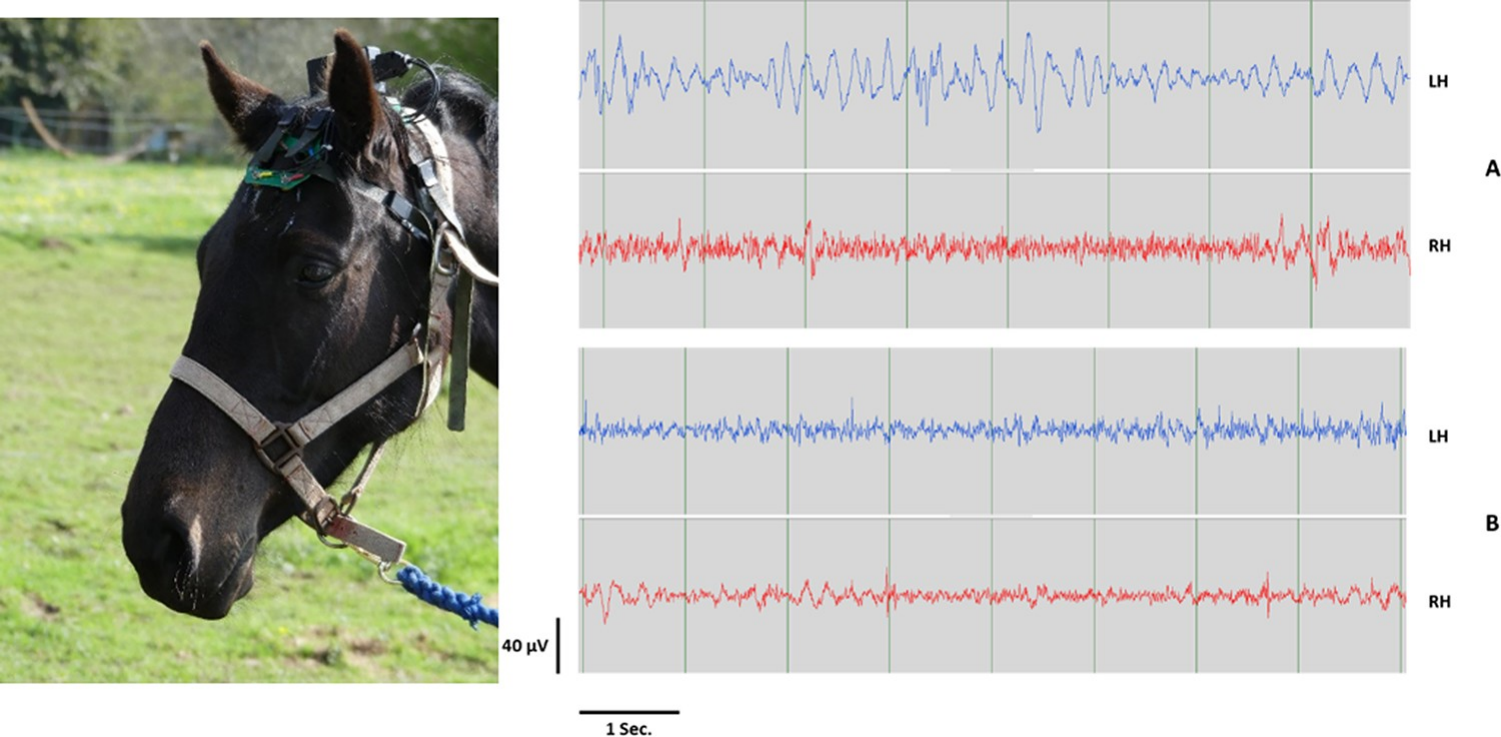
(left) EEG headset used in the present study. (right) Example of 8 sec. electroencephalography (EEG) recordings of left (LH) and right (RH) hemispheres obtained with the EEG headset after removing artefacts in both horse populations (A- recreational horse; B riding school horse). LH wave for recreational horses are characteristic of slow waves.

The horses’ electrophysiological activity was recorded using an ambulatory EEG headset (patent # R23701WO). This headset allows an easy and fast positioning of 5 electrodes on the horse’s forehead over the frontal and parietal bones [31] (Fig 1). The electrodes were located on each side of the horse’s forehead allowing the recording of the differential activity between the most occipital part of the brain and the most frontal one of the left and right hemisphere separately [31] and were filled with a conductive gel (NEURGEL250F Spes Medica) in order to ensure an electrical contact between the electrodes and the horse’s head. The ground electrode was placed on the back of the left ear. The system was completed by a telemetric EEG recorder made by RF-TRACK (Cesson-Sevigne, France). The EEG amplifier, based on a Texas Instruments integrated circuit ADS1294, was connected to a Bluetooth transmitter allowing to check in real time the EEG recording quality and to an onboard micro SD card which allowed keeping a recording of the raw data during the experiments. The whole telemetric recording setup weighing 110g was fixed on the headset.

For a week before the beginning of the recordings, horses were trained daily to wear the non-invasive EEG headset (*i*.*e*. 5min per day). Horses show rapid changes in attentional shift and changes of state have been shown, in awake horses, around every 3–30 sec. [40, 42, 43] where horse starts changing position, moving head or ears [42–44]. Therefore, in order to enhance chances to have a reliable measure, we performed 30 sec. recording sessions when the horse was being held in absence of any artificial stimulation (*i*.*e*. no visual, auditory or tactile artificial stimulation), ensuring that it was quiet standing all along the procedure [40]. During the recordings, horses were handled by one of two unfamiliar experimenters (Sd’I or MH) in the familiar arena, standing 1m from the horse, arms along the body and looking to the ground. Both experimenters handled randomly recreational and riding horses in order to avoid any experimenter effect. Two EEG recordings sessions took place spaced two days apart.

The EEG recordings were then processed offline using a software “EEGReplay4.3” developed by RF-TRACK. Before the EEG analysis, the large artifacts due to movements (body, head, ears) were automatically removed using a homemade software with Python3.6.4 environment. In order to prevent any contamination from muscle artifact, we first used a smoothly Savitzky Golay function integrated in Python3.6.4. The smoothed signal was then subtracted from the raw signal to remove the large low frequency artifacts. To achieve this removing artefact process, the signal variations with amplitudes larger than 200 μV were excluded [45]. Further examination for the data showed that the maximum good quality recording duration common to all horses (in the same behavioural state) was 8 sec. Therefore, since there were no significant differences in the EEG profiles between the two sessions (Wilcoxon test, N = 18, Left Hemisphere: 42<V<96, 0.10<p<0.57; Right Hemisphere: 36<V<80, 0.18<p<0.89) and intra-individual reliability proved high (see results), we kept for further analysis and inter-individual comparisons the 8 seconds best quality recordings of the first session.

Once the artifacts were removed, a Fast Fourier Analysis was then performed every 4 mSec on a 1 Sec. window. Then, using “EEGReplay4.3” RF Track software, proportions of the different EEG waves (delta (δ: <4 Hz), theta (θ: 4–8 Hz), alpha (α: 8–12 Hz), beta (β: 12–30 Hz) and gamma (γ: >30 Hz)) were automatically calculated from the total EEG power spectrum for both the right and the left hemisphere independently (see also [40]). Wave frequency proportions data considered for the subsequent analysis corresponded to the median proportion of the power values extracted over the continuous recording (e.g. for 8 sec.: (250x0.004s)*8 = 2000 measures).

### Back state evaluation

#### Neck shape measurement

Precise measures of both neck shape and the spine state (through practitioner examination and EMGs evaluations) have shown that a hollow or a flat neck reflects muscular tensions in different parts of the spine, while a round neck characterizes healthier backs [22]. Neck shape can easily be estimated by looking at the angle formed by the segment linking the cervic-thoracic junction and the trapezium cervical ligament at C3 with the segment linking the trapezium ligament and the dorsal part of the atlas. According to Lesimple et al [22]’s measures, the horses’ neck was classified as hollow, flat or round (*i*.*e*. the horse was handled by an experimenter, standing in pasture for recreational horses, in stable for riding horses). Since flat and hollow necks tend to reflect an altered spine state, the horses were classified as having a round *versus* hollow/flat neck. This classification was performed following Lesimple et al 2012’s methodology [22], by two observers (MS and MH), with 94.4% concordance (Kappa = 0.89, p<0.0001) independently and before the EEG profiles were analyzed.

#### Static surface electromyogram [22]

The sEMG examinations were conducted separately by one experimenter (C.L.), blind to the results of the EEG and neck shape data, using a wire free device (Myovision®). The experimenter had 2 joysticks with 5 electrodes on each, designed to record muscular activities at the level of the vertebrae before and after the joystick location. Muscular activities recorded were relayed to a computer coupled with a receptor. The two joysticks were placed at the level of C2, C6, T3, T9, T17, and L6 (Fig 3) one on each side of the spine, and electrodes gave the muscular activities at the level of C1, C3, C5, C7, T1, T3, T8, T10, T16, T18, L5 and S1. Thus we obtained muscular activity all along the neck, at the level of the shoulder, at the basis of the withers, at the level of the thoracolumbar joint and at the level of the lumbosacral joint, which are reported in the literature as very likely to be affected by musculoskeletal lesions [46]. In the two populations, examinations were performed on a flat ground, outside the pasture in the recreational group, and in the corridor of the stable in front of each horse’s box in the second group, in absence of any noise or disturbance (working activity, people around…). The experimenter paid attention to the horses’ limb positions: forelimbs and hind limbs were on a line (*i*.*e*. standing square with the hind hoof directly behind the fore hoof). As the assessment of horses sEMG using Myovision® device was shown to be highly repeatable [22], each horse underwent a single evaluation. Horses were kept motionless, slightly restrained with a rope, by another experimenter (M.S.). At the time of sEMG measures, 5 horses were not available anymore, which restricted the sample size for this measure to 13 horses.

Muscular activities were recorded along the spine (312 tested sites: 13 horses * 12 vertebral sites *2 sides of the spine) and the raw sEMG values were used (μV). In order to evaluate the global state of individuals’ backs, we calculated the muscular activity’s median value of all sites along the spine per horse (24 tested sites: 12 vertebral sites*2 sides). Finally, in order to assess the degree of asymmetry we calculated the difference between the left and the right sides. Thus we obtained a median value of the global asymmetry along the spine per individual.

### Observations of stereotypic behaviours

All observations and tests were performed by the same trained experimenter (M.S.) during week days and generally during the quiet hours (*i*.*e*. outside teaching activities for riding school horses), before the two EEG recordings sessions were performed. A microphone (Sony1ECM-T6) was used to record the experimenter's voice, giving individuals' information and observations. Six 10-min observation sessions (*i*.*e*. 60 min in all per horse) were performed when riding school horses were in their home stall and when leisure horses were in their own pasture. Observations were made during three time periods (twice for each time period): morning (10:00–12:00am), afternoon (2:00–5:00pm) and 30 min before meals (*i*.*e*. 9:30am and 6:00pm here, favourable for observing abnormal repetitive movements in restricted conditions, but that did not correspond to any particular feeding time for leisure horses). Sampling was on an all-occurrence basis, with half of the horses being observed simultaneously, which means that the behaviours concerned were recorded each time they occurred and for each horse.

Data recorded were the numbers (per hour) of stereotypic behaviours and other abnormal repetitive behaviours displayed by each individual (see Table 2).

**Table 2:** Type, classification and description of recorded abnormal repetitive behaviours.

SB/ARB behavioural description	Type
head tossing / nodding: vertical movements of head and neck	motor
striking with forelimb: horse hits the door or wall with one of its forelegs	motor
box walking: repetitive tracing a route within the stable	motor
cribbing / wind sucking: the horse grasps a fixed object with its incisors, pulls backwards and draws air into its oesophagus	oral
head movements (other than head tossing / nodding): movement of the head	motor
lip shivering: shivering of the lower lip	motor
repetitive displacement of the saddle support on the stall door	motor
tongue movements: movements of tongue, inside or outside its mouth	oral
repetitive biting: biting a given object in its environment	oral
repetitive licking: licking a given object in its environment	oral

### Statistical analysis

As data were not normally distributed, we used non-parametric tests [47].

In order to study the intra-individual variability between the two EEG recording sessions, we ran first Wilcoxon tests to verify that there were no difference at the populational level between both sessions and then Spearman correlation tests to examine whether individual EEG profiles (*i*.*e*. proportion of each wave type in each hemisphere, N = 10 (delta Right (R), theta R, alpha R, beta R, gamma R, delta Left (L), theta L, alpha L, beta L, gamma L)) were stable over time at the individual level. That showed no significant difference (N = 18, Left Hemisphere: 42<V<96, 0.10<p<0.57; Right Hemisphere: 36<V<80, 0.18<p<0.89), and considering that a previous study showed that horses have consistent power profiles over days [31], only recordings from the first session (with data for more horses since three horses could not be tested the second time for practical reasons) were kept for the subsequent analysis. As horses were trained daily during a week before the experiment to wear the EEG device, we assume that no habituation happened between the first and the second session given that horses were already habituated before the first session. Mann Whitney tests were conducted to compare wave frequency proportions according to the horses’ neck shape (*i*.*e*. flat/hollow versus round), for the left and the right hemisphere independently, as well as the muscular tension along the spine according to the horse’s neck posture. Spearman’s correlation tests, including fdr (*i*.*e*. false discovery rate) corrections that automatically adjust p values according to the number of tests performed (α = 0.05), were additionally performed to test relationships between wave frequency proportions measured in each hemisphere and the horses’ sEMG measurements on one hand and stereotypic behavior on the other hand. Spearman correlations were additionally run between wave frequency proportions and the muscular activity recorded for each specific vertebral site on one side and on the other side. The mean value of the left and right sides was considered in that case for each vertebral site. The relationship between the horses’ age and the EMG measures on one side and EEG measures on the other side have been tested using Spearman correlation tests. Finally, sex effect was not tested considering that only two stallions were included in the population. Indeed, they can not be grouped with geldings since they can behave differently.

All statistics were performed with R v. 3.5.0 (The R foundation for statistical computing, http://www.r-project.org/).

## Results

In accordance with a previous study [31], we found that the horses had highly consistent individual EEG profiles over time with 11 horses showing high (0.64≤r≤0.74) and two moderate (0.52≤r≤0.57) correlations in their individual EEG profiles (proportion of each wave type in each hemisphere) over the two sessions (Table 3). Only two horses showed a lower correlation. Three horses (S2, S4, G4) were not tested at the second session for practical reasons. For further analysis and to enhance conformity in measures between horses, only recordings from the first session were thus kept for the subsequent analysis.

**Table 3:** Individual EEG profiles correlations between the two recording sessions (Spearman correlations).

Individuals Pop.1	p	r	Individuals Pop. 2	p	r
S1	NA	NA	G5	0.08	0.57
S2	**0.02**	0.70	G6	NA	NA
G1	0.75	-0.1	G7	**0.04**	0.64
G2	NA	NA	G8	**<0.001**	0.93
G3	**0.02**	0.72	G9	**0.004**	0.84
G4	0.12	0.52	M4	**0.009**	0.79
M1	**0.02**	0.73	M5	**0.01**	0.76
M2	0.23	0.41	M6	**0.0004**	0.91
M3	**<0.001**	0.97	M7	**0.0008**	0.90

Mean waves proportions (delta Right (R), theta R, alpha R, beta R, gamma R, delta Left (L), theta L, alpha L, beta L, gamma L) recorded for the first and second session were correlated per horse. Significant values are in bold.

EEG activities recorded for the 18 horses during the first session are shown Table 4.

**Table 4:** EEG activity recorded over the first session on the 18 horses.

EEG wave activity (median proportions, %)	Delta (LH)	Theta (LH)	Alpha (LH)	Beta (LH)	Gamma (LH)	Delta (RH)	Theta (RH)	Alpha (RH)	Beta (RH)	Gamma (RH)
Min	0.03	4.2	2.6	10.6	3.7	0.1	6.7	3.4	8	5.3
Max	28.5	59.1	19.3	52.6	46	27	67.1	20.4	44.4	40.7
μ	6.6	23.5	9.6	30.9	23.7	4.9	32.4	10.6	27.4	20.1
sd	8	12.9	3.9	10.2	12.4	7.7	17	4.3	10	10.9

**Fig 2:**
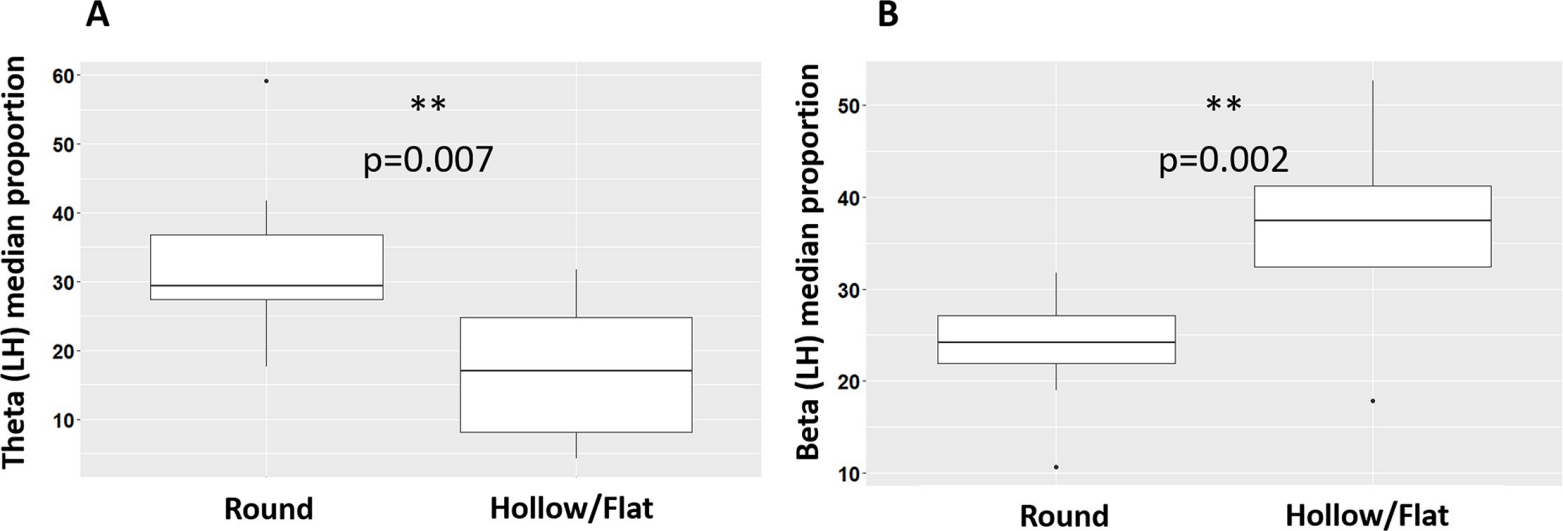
Relation between EEG power profile and postural indicators of spine state: median theta **(A)** and beta **(B)** proportion recorded in the left hemisphere according to the horse’s neck shape (as measured following Lesimple et al [22]’s method). Mann-Whitney test. ***p<0.001; **p<0.01; *p<0.05.

EEG activity was characterized by more theta waves (Mann Whitney test, W = 70, p = 0.007) and less beta waves (W = 8, p = 0.002) in the Left Hemisphere (LH) in horses with a round neck (N = 9) compared to horses with a hollow/flat neck (N = 9) (Fig 2).

Through practitioner examination and sEMG evaluations, Lesimple et al. [22] have shown that muscular tensions in different parts of the spine were associated with a hollow or flat neck. This was confirmed here since horses with a round neck had a higher number of vertebral sites with less than 1μV (*i*.*e*. vertebrae not affected) than horses with a hollow/flat neck (Mann-Whitney test, N = 13, W = 32.5, p = 0.05). Thus, it appears that the healthier horses with a round neck produced more theta waves. No difference was found in the right hemisphere nor for any other frequency bands.

**Fig 3:**
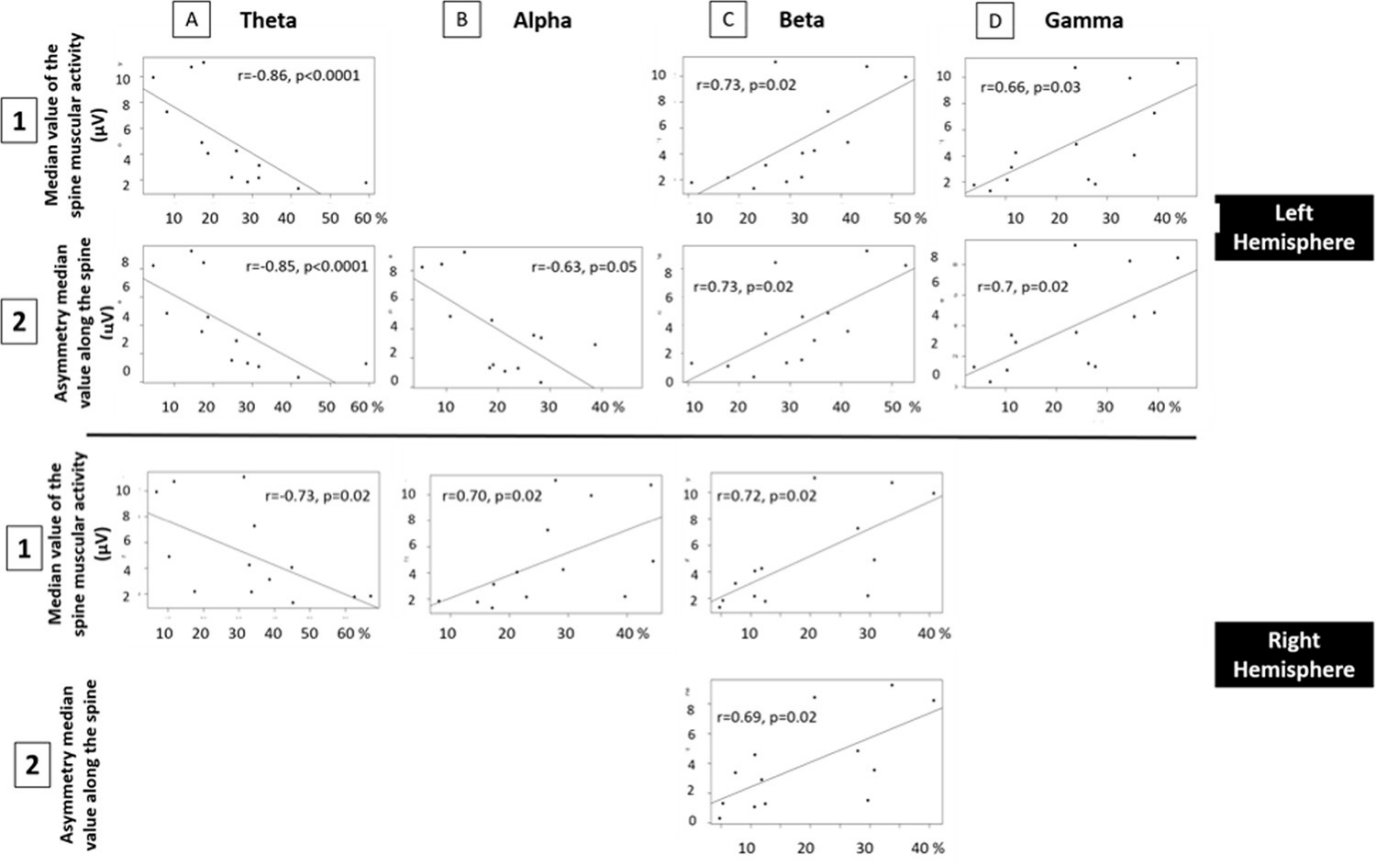
Correlations between the proportions of the different waves in the quantitative resting- state EEG in each hemisphere and sEMG measures along the spine. **1** median value of the spine muscular activity; **2** the asymmetry median value along the spine. Only significant correlations are shown.

This relation between spine state and EEG power profiles was further and still more convincingly confirmed by the sEMG data obtained from 13 horses (Fig 3).

In both hemispheres, the median value of the spine muscular activity was negatively correlated with theta and, like the median asymmetry, positively correlated with beta and gamma relative frequencies. Thus, the more muscular tensions and asymmetry along the spine, the higher the beta and gamma activity and the lower the theta activity. This was still more so when only the left hemisphere was considered with less slow (theta and alpha) and more fast (beta and gamma) waves activity when the asymmetry was higher.

**Fig 4:**
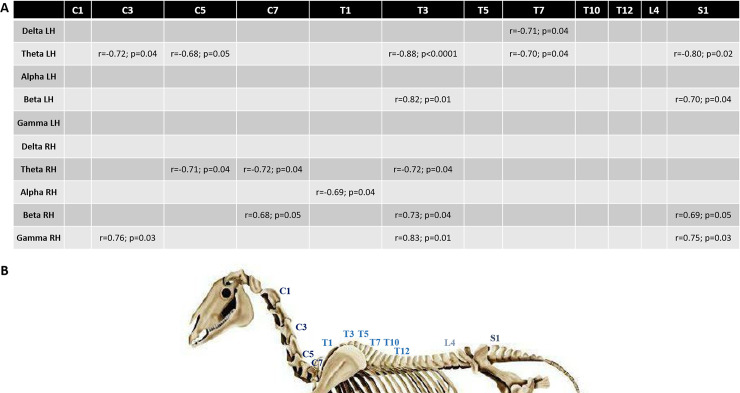
Relationship between resting state EEG power profile and sEMG measures at different sites along the horse’s spine. **(A)** Correlations between the proportion of each wave type in the left (LH) or right (RH) hemisphere and sEMG measures at each spine site; **(B)** representation of the different sEMG recording sites along the spine (C = cervical, T = thoracic, S = sacral).

Finally, these findings were reflected at the level of the precise spine sites, as in all cases, more muscular tension was reflected by more fast waves and less slow waves. The correlations were particularly clear at the level of T3, a particular vertebral site where the two muscle fascia responsible for head/neck movements join and at S1, the sacral area (Fig 4).

It is worth noting that no correlations were observed between either sEMG or EEG measures and the horse’s age (Spearman correlation, -0.18<r<0.57; p>0.05).

The muscular tension along the spine (*i*.*e*. sEMG median value) was also correlated with the number of stereotypic behaviours performed per hour in their home environment (N = 13, N Spearman test, rs = 0.67, p = 0.01). Thus, the more tension along the spine, the more the horses may have tried to cope with the suggested associated pain [18, 25, 32]. Moreover, the EEG measures indicated that the more stereotypic the horse was, the less theta waves were recorded in its left hemisphere (N = 13, rs = -0.58, p = 0.05) as there were correlations between the measures of muscular activity along the spine and theta waves. Interestingly the correlation between muscular activity along the spine and stereotypic behaviours was maintained when only motor stereotypies (rs = 0.67, p = 0.01) were considered. It was not possible to test the correlation for oral stereotypies as only three horses performed these behaviours. Similarly, the lower the number of motor stereotypic behaviours the higher theta proportion in the left hemisphere (rs = -0.59, p = 0.04), confirming the close relationship between physical disorders and brain production.

## Discussion

The clear correlations observed here between muscular activity along the spine, its asymmetry, neck shape alterations and increased fast (beta and gamma) waves, suggest strongly the involvement of fast waves in the processing of spontaneous chronic pain in this species. This involvement of fast waves is concordant with the finding that massage therapy suppresses the beta rhythm in low back pain human patients [48] and with the association between ongoing pain intensity and amplitude of beta and gamma waves in patients suffering chronic back pain [4]. The fact that horses presenting higher frequency bands in their resting state (*i*.*e*. quiet standing) EEG were also performing more stereotypic behaviours indirectly confirms that these horses were experiencing a painful condition, as they were, like humans experiencing chronic pain, performing an “addictive” behaviour [1]. The presence of fast waves might indicate that they may not be able to experience real quiet states, maybe because of a persistent pain. In contrast, healthier horses presented more theta waves, which also confirm the major interest of theta waves when considering quantitative EEG. However, recent studies found an increased power amplitude in theta in patients with neurogenic pain and in rodents experimentally subjected to chronic constriction injury [3]. This may be due to the type of pain, as neurogenic pain may be involving a more noxious/subacute component than chronic back pain, potentially involving a more emotional component [1], in which case it would be important to look for different biomarkers (*i*.*e*. neurophysiological components obtained through neuroimaging [49] according to the source of pain. One other explanation could be in the measures of the noxious stimuli applied (objective aspect) versus the perceived (by the individual) pain as opposed patterns in the predominant types of frequency bands, with fast waves being positively correlated to ongoing and chronic subjective pain, assessed by human subjects [4, 7, 8]. Slow waves and in particular theta have otherwise been associated with reduced anxiety [5], positive and calm emotions [50] and attention [51]. Chronic pain is associated with negative affect and lowered attentional engagement both in humans [52] and animals [23, 53]. Horses with a healthier back exhibit more positive behaviours towards an experimenter [17, 25]. Taken together, all these aspects suggest that a lowered theta power may reveal the subjective perception of spontaneous chronic pain by horses. The correlation between the theta versus beta waves and the muscular activity measures at the different levels of spine reinforces this association between EEG power profile and back tension, especially as the clearest correlations were at T3, a thoracic vertebral site often involved with vertebral problems such as overlap of dorsal spinuous processes [19] and central for the head-neck positioning. However, our study confirms once again that age has a low impact on horse welfare and pain [54].

Although correlations between muscular tension along the spine and EEG activity were observed regarding brain production both in the right and left hemispheres, a particular association was noticed between horses’ back health and the left hemisphere activity. Indeed, horses which showed higher production of theta waves in the left hemisphere were those that had more a round neck and performed less stereotypic behaviours per hour. It is probable that these horses, which do not experience back pain, have also a more positive assessment of their life conditions, which would be in accordance with the known particular involvement of the left hemisphere for positive processing [55–57].

These results are all the more remarkable as they rely upon short recordings and thus suggest that EEG power profiles could be of major interest for assessing the current animal’s subjective state beyond the objective measure of back tension. In order for this tool to be used more widely, recording conditions (*i*.*e*. the closer to the horse’s home environment) and durations (*i*.*e*. longer recordings according to questions) still need to be refined, but these findings open new lines of research in the comparative study of back disorders, the potential chronic pain associated and its impact on cognitive functions. Studies on a large panel of species would be an important way of clarifying these issues and promising useful tools.

## Supporting information

S1 File (XLSX)
